# Diving behavior in a free‐living, semi‐aquatic herbivore, the Eurasian beaver *Castor fiber*

**DOI:** 10.1002/ece3.3726

**Published:** 2017-12-12

**Authors:** Patricia Maria Graf, Rory Paul Wilson, Lea Cohen Sanchez, Klaus Hacklӓnder, Frank Rosell

**Affiliations:** ^1^ Institute of Wildlife Biology and Game Management University of Natural Resources and Life Sciences Vienna Austria; ^2^ Department of Natural Sciences and Environmental Health Faculty of Technology, Natural Sciences and Maritime Sciences University College of Southeast Norway Telemark Norway; ^3^ Biosciences College of Science Swansea University Swansea UK; ^4^ Institute of Geography School of Geoscience University of Edinburgh Edinburgh UK

**Keywords:** *Castor fiber*, diving depth, Eurasian beaver, herbivore, semi‐aquatic, VeDBA

## Abstract

Semi‐aquatic mammals have secondarily returned to the aquatic environment, although they spend a major part of their life operating in air. Moving both on land, as well as in, and under water is challenging because such species are considered to be imperfectly adapted to both environments. We deployed accelerometers combined with a depth sensor to study the diving behavior of 12 free‐living Eurasian beavers *Castor fiber* in southeast Norway between 2009 and 2011 to examine the extent to which beavers conformed with mass‐dependent dive capacities, expecting them to be poorer than wholly aquatic species. Dives were generally shallow (<1 m) and of short duration (<30 s), suggesting that the majority of dives were aerobic. Dive parameters such as maximum diving depth, dive duration, and bottom phase duration were related to the effort during different dive phases and the maximum depth reached. During the descent, mean vectorial dynamic body acceleration (VeDBA—a proxy for movement power) was highest near the surface, probably due to increased upthrust linked to fur‐ and lung‐associated air. Inconsistently though, mean VeDBA underwater was highest during the ascent when this air would be expected to help drive the animals back to the surface. Higher movement costs during ascents may arise from transporting materials up, the air bubbling out of the fur, and/or the animals’ exhaling during the bottom phase of the dive. In a manner similar to other homeotherms, beavers extended both dive and bottom phase durations with diving depth. Deeper dives tended to have a longer bottom phase, although its duration was shortened with increased VeDBA during the bottom phase. Water temperature did not affect diving behavior. Overall, the beavers’ dive profile (depth, duration) was similar to other semi‐aquatic freshwater divers. However, beavers dived for only 2.8% of their active time, presumably because they do not rely on diving for food acquisition.

## 1 INTRODUCTION

Mammals evolved on land (Kemp, 1980, 2005) and were consequently under selection pressure to operate efficiently in an air‐based medium. Some mammal taxa, however, secondarily returned to the water, a medium that is denser and has a higher thermal conductivity than air. This has profound consequences for movement and heat loss (Williams, 2001) and involves a plethora of pressure‐linked problems (Kooyman & Ponganis, 1998). In homeotherms, marine mammals possess some of the most extreme adaptations, including thick insulation, high streamlining, a reduced surface area, planar limbs for propulsion, and a suite of features associated with dealing with apnea and pressure (Berta, Sumich, & Kovacs, 2015; Fish, 1994). These extreme specializations are possible because fully, or almost fully, aquatic mammals need only to operate in water. Through this, marine species are able to dive to depths of up to 2992 m and for durations of up to 137.5 min (in Curvier's beaked whale *Ziphius cavirostris*; Schorr, Falcone, Moretti, & Andrews, 2014).

Semi‐aquatic mammals spend the major part of their life operating in air but are apparently reliant on water (Dunstone, 1998). Living in two fundamentally different media precludes these animals from being particularly well adapted for either environment (Fish, 2000; Williams, 1999). The thick, air‐filled fur or plumage of semi‐aquatic species provides valuable insulation but makes these animals positively buoyant and, thus, increases upthrust and energy use during submergence (Butler & Jones, 1997). Moreover, their drag‐based propulsion with paddles is less efficient and uses more energy than the lift‐based propulsion with hydrofoils employed by fully aquatic mammals (Fish, 1994). Perhaps, as a result of this, and in any event due to their less specialized morphology and their prevalent use of freshwater habitats, semi‐aquatic species are generally limited to dive at shallow depths (Fish, 2000). However, some aspects of diving in semi‐aquatic species do conform to those of more specialized divers, for example, dive durations generally tend to increase with diving depth and body mass (Halsey, Blackburn, & Butler, 2006; Schreer, Kovacs, & O'Hara, 2001), while deeper dives are typically accompanied by steeper descent and ascent angles (Lydersen, Martin, Gjertz, & Kovacs, 2007; Ropert‐Coudert, Grémillet, & Kato, 2005).

Small mammalian freshwater divers are little studied though (Hays et al., 2007), with most research being conducted in captivity and focusing on physiological aspects of diving (e.g., Dunstone, 1993; MacArthur, 1984). A common finding of such studies is that semi‐aquatic species face increased energetic costs during diving, mainly due to higher thermoregulatory needs (MacArthur & Krause, 1989), particularly in cold water (MacArthur, 1984), as well as increased physical effort needed to overcome buoyancy and drag (Fish, Smelstoys, Baudinette, & Reynolds, 2002; Williams, 1983). Fortunately, small, high‐precision pressure transducers are now being increasingly used to quantify the diving behavior of free‐living, semi‐aquatic species, providing insight into the diving abilities of these animals (e.g., Harrington et al., 2012; Hays et al., 2007) and even movement‐based power use during diving (using “Overall‐” or Vectorial Dynamic Body Acceleration, ODBA or VeDBA, c.f. Wilson et al., 2006; Stothart, Elliott, Wood, Hatch, & Speakman, 2016). For example, work on American mink *Neovison vison* demonstrated extensive diving during winter, and the authors were speculating that the energetic gains of aquatic foraging should outweigh thermoregulatory costs (Bagniewska, Hart, Harrington, & Macdonald, 2013; Bagniewska et al., 2015; Harrington et al., 2012). In another elegant study, Bethge, Munks, Otley, & Nicol, 2003 found that dives in platypus *Ornithorhynchus anatinus* appeared to be mainly within their estimated aerobic dive limit and that recovery times followed Kramer's optimal breathing theory (Kramer, 1988).

We studied the diving behavior of a free‐living, amphibious herbivore, the Eurasian beaver *Castor fiber*. Both the Eurasian and the North American beaver *Castor canadensis* are nocturnal, monogamous, semi‐aquatic rodents. Beavers are highly territorial, live in family groups, and occupy lodges or bank dens along freshwater bodies (Wilsson, 1971). Dams are built to, among other things, raise water levels and hold functional winter food caches (Hartman & Törnlöv, 2006). Beavers have a preference for willows *Salix* spp. and poplars *Populus* spp. (Haarberg & Rosell, 2006) but also forage on aquatic plants (Parker, Caudill, & Hay, 2007).

The beavers’ fusiform body with short limbs, webbed hind feet, and waterproof fur reflects the animal's adaption to an amphibious life (Allers & Culik, 1997). In fact, it has been suggested that beaver streamlining is similar to that of fully aquatic animals such as phocid seals (Reynolds, 1993). In beavers, physiological adaptions to diving include bradycardia and a minor postdive tachycardia (Clausen & Ersland, 1970; Swain, Gilbert, & Robinette, 1988). However, beavers feature much lower myoglobin levels than fully aquatic species (Mirceta et al., 2013) and reduced water temperatures have been shown to diminish their swimming time (Nolet & Rosell, 1994) and increase their thermoregulatory costs during immersion (MacArthur & Dyck, 1990).

However, to date, diving in beavers has been exclusively studied in *C. canadensis* and primarily in captive settings (but see MacArthur & Dyck, 1990) and concentrated on physiological and morphological aspects of diving (Allers & Culik, 1997; Clausen & Ersland, 1968, 1970; MacArthur & Dyck, 1990; Swain et al., 1988). By contrast, behavioral aspects of diving in free‐living beavers are poorly studied; research on diving depths is generally lacking, while studies reporting dive durations are of anecdotal nature (<1 min reported in Clausen and Ersland (1970), 15 min reported in Irving and Orr (1935), 22.75 ± 2.78 min observed by MacArthur and Dyck (1990), although in this study beavers likely exploited air pockets beneath the ice).

We deployed tri‐axial accelerometers combined with depth transducers on dominant Eurasian beavers to study dive parameters including maximum diving depth, dive duration, bottom phase duration, and the mean number of dives per night. Being positively buoyant, beavers face increased mechanical costs during submergence (Fish et al., 2002). Thus, we hypothesized that maximum diving depth and both total dive and bottom phase duration would be linked to the physical effort during diving measured via mean VeDBA and vertical velocities during descent and bottom phases. Moreover, as water temperature is predicted to have a radical effect on heat loss, which has to be counteracted by increasing power, and thereby, increasing oxygen use (Ciancio, Quintana, Sala, & Wilson, 2016), we would expect dive capacity to be compromised by low water temperatures. As a result, we might expect semi‐aquatic animals, which are not obliged by their lifestyle to dive excessively, to reduce the incidence of diving in cold conditions. We thus explored differences in dive frequency per night with respect to water temperature. Based on the semi‐aquatic nature of beavers, we predicted that (1) they would primarily execute short and shallow dives, (2) that deeper dives would be linked to lower mean VeDBA and higher vertical velocities during the descent, and (3) that dive duration would decrease with higher mean VeDBA during the descent. Furthermore, we expected reduced buoyancy forces with depth due to compression of body‐associated air leading to lower rates of oxygen consumption (Wilson, Hustler, Ryan, Burger, & Noldeke, 1992) and thus predicted (4) longer bottom phase durations during deep dives. Finally, because water temperature modulates energy expenditure so notably in beavers (MacArthur & Dyck, 1990), we predicted (5) that beavers should simply dive less with increasingly cold water temperatures. This work examines the diving behavior of beavers in light of these hypotheses and attempts to interpret them with respect to behavioral mechanisms for increasing diving efficiency before putting the mass‐specific diving capacities of beavers into a broader diving endotherm perspective.

## 2 MATERIALS AND METHODS

### 2.1 Study area and animals

The study was conducted in spring and autumn between 2009 and 2011 in Telemark County, southeastern Norway (59°23′ N, 09°09′ E). The study sites are located at three large rivers, the Straumen, Gvarv, and Sauar, which all empty into Lake Norsjø. The river sections are mostly slow flowing with stable water levels, about 20–150 m wide (Campbell, Nouvellet, Newman, MacDonald, & Rosell, 2012) and on average approximately 20 m deep (min. 2 m, but typically between 4 and 10 m; source: Statens Kartverk Telemark). The three rivers feature similar depth structures and are deep and wide enough to make damming for beavers unnecessary. Riverbanks are dominated by semi‐agricultural and riparian woodland structures with tree species such as gray alder *Alnus incana*, willow *Salix* spp., bird cherry *Prunus padus*, common ash *Fraxinus excelsior*, rowan *Sorbus aucuparia*, birch *Betula* spp., and Norway spruce *Picea abies* (Haarberg & Rosell, 2006). Man‐made impoundments (Straumen) and river sections that widen up to natural lakes (Gvarv and Sauar) lead to reduced ice cover in winter (Campbell et al., 2012). The climate in the area is cool continental with a mean annual temperature of 4.6°C and a mean annual precipitation of 790 mm (Campbell et al., 2012). Water temperatures differed considerably between the seasons (*H* = 2435.6, *df* = 1, *p* < .001), with colder water temperatures during spring ($\overset{¯}{x}$ ± SD = 3.6 ± 0.8°C; April–May) and warmer water temperatures during autumn (9.7 ± 1.4°C; September–November). The earliest evidence for Eurasian beavers in the area is from the 1920s (Olstad, 1937); today, the population is at carrying capacity (Graf, Mayer, Zedrosser, Hackländer, & Rosell, 2016; Steyaert, Zedrosser, & Rosell, 2015). Both hunting pressure and the presence of natural predators in the area were low (Graf, Mayer, Zedrosser, Hackländer, & Rosell 2016). Since 1997, beavers in the study area have been monitored every year as part of a long‐term capture–mark–recapture study (Campbell, Rosell, Nolet, & Dijkstra, 2005; Campbell et al., 2012). Dominance status and sex of each beaver used in the study were previously determined (for methods see Rosell & Sun, 1999; Campbell et al., 2012).

We captured 21 dominant Eurasian beavers from 16 different territories between 7:00 pm and 7:00 am with a landing net from a boat (Rosell & Hovde, 2001). The animals were transferred into a cloth bag, where they were tagged without the need for anesthesia. Average handling time was 30 ± 5 min. We used two‐component epoxy resin to attach tags consisting of a VHF transmitter (18 × 35 mm, 10 g; Reptile glue‐on series R1910; Advanced Telemetry Systems, USA) and a data logger recording pressure and tri‐axial acceleration (15 × 90 mm, 62 g; JUV Elektronik, GER) onto the fur of the lower back along the spine, 15 cm above the scaly tail. The units were connected with wire or glued together and integrated in half‐mesh net covering. The whole unit was 130 × 90 mm in size (incl. netting) and weighed 90 g in air, which amounts to 0.46% of the body mass of the lightest beaver (19.5 kg) used in this study. We measured body length and weighed beavers before releasing them at the trapping site. After 2–3 weeks, the animals were recaptured via VHF telemetry, and the tags were cut out of the fur with a scalpel. This procedure only affected the guard hairs, leaving the under‐fur unaffected (Graf, Wilson, Qasem, Hackländer, & Rosell, 2015). Data from nine of the tags were not used in the analysis, as three units failed to log data (because their memory cards popped out of their housings), three units had corrupt readings (which could be attributed to gnawing marks on the sensors), and three units recorded only between 1 and 3 nights of data (which was also accompanied by gnawing marks and water intrusion into tag housings, or early tag loss during molting in spring).

### 2.2 Data preparation and dive analysis

The data logger used in this study recorded pressure (range 950–10,000 mB) and tri‐axial acceleration (±4 g) with 22‐bit resolution. Data were recorded at a frequency of 1 Hz for pressure and 8 Hz for acceleration in three orthogonal axes corresponding to the beaver's surge (longitudinal), heave (dorso‐ventral), and sway (latitudinal) axes and stored on a 1 GB memory card. Pressure data were used to identify maximum diving depth, dive duration, dive phases and, in particular, bottom phase duration via points of inflection in the dive profile. Tri‐axial accelerometers record posture (static acceleration) and movement (dynamic acceleration) (Shepard et al., 2008) with respect to the earth's gravitational field (1 *g *=* *9.81 m.s^−2^) and can thus be used for behavioral identification in animals (e.g., beavers; Graf et al., 2015). The accelerometers were used to derive mean VeDBA according to methods described in Qasem et al. (2012), which been shown to be a good proxy for the rate of oxygen consumption and therefore power use (Qasem et al., 2012). Accelerometry data were also used to derive principal activity periods PAPs (time from emergence from the lodge to return, see also Graf, Hochreiter, Hackländer, Wilson, & Rosell, 2016) to evaluate the relative time spent diving per night.

All dive events were visually inspected and exported using Multitrace (Jensen Software Systems, GER). We used zero‐offset correction (ZOC) to correct for sensor drift in the pressure readings (Hagihara, Jones, Sheppard, Hodgson, & Marsh, 2011), which we adjusted manually if needed. We selected a dive threshold of 0.3 m to ensure pressure readings indicating dives did not result from wave action or the animal simply angling its back down into the water. Descent, bottom, and ascent phases were automatically recognized and delineated by the program but could be adjusted manually.

We standardized all datasets to range from 5 to 7 full nights, excluding data from the capture night to account for possible tagging effects (Graf, Hochreiter, et al., 2016). Water temperature data for the Gvarv and Straumen rivers were provided by the Norwegian Water Resources and Energy Directorate (NVE). For the Sauar River, no temperature data were available for the lower reaches of the river where we captured beavers. We thus used temperature data from the Gvarv River for the Sauar, as both rivers are in the same area (approx. 10 km linear distance apart), are of similar size, and feature similar flow characteristics (personal communication, Å. Kvambekk, NVE). Daily mean water temperature for the rivers was averaged over the week of tag deployment.

### 2.3 Statistical analysis

We used linear mixed‐effects (LME) models for the response variables “maximum diving depth” and “dive duration” and included the covariates “mean VeDBA during descent,” “vertical velocity during descent,” “water temperature,” “sex,” and “mass” in both models. In addition, we created a separate LME without covariates to analyze the relationship between “maximum diving depth” and “dive duration,” two variables which are known to be interrelated and which are commonly investigated in other diving homeotherms (e.g., Halsey, Butler, & Blackburn, 2006; Harrington et al., 2012). We used a generalized linear model (GLM) for the response variable “mean number of dives per night” and included “water temperature,” “sex,” and “mass” as covariates. The covariate “sex” was included to determine whether beavers have sex‐specific nutritional needs (aquatic plants), while “mass” was included to determine whether heavier beavers were less buoyant during submergence. The response variables “maximum diving depth,” “dive duration,” and “mean number of dives per night” were ln‐transformed to normalize residuals.

To analyze bottom phase duration, we used a double‐hurdle model (Cragg, 1971), based on the assumption that individuals decide (i) whether to invest in a bottom phase or not, and whether there is a bottom phase, (ii) how long this bottom phase should be. We used a negative binomial model for the binary response variable “bottom phase” (0 = “no bottom phase” and 1 = “a bottom phase”) and a generalized linear mixed model (GLMM) with a log‐link function to model the actual continuous response “bottom phase duration.” We included the predictor variables “maximum diving depth,” “water temperature,” and “mean VeDBA during the bottom phase” in the double‐hurdle model. In all models, we used “individual” as random effect to account for individual variation in diving performance. Initially, we also nested “individual” within “year,” but then omitted “year” as results were essentially unchanged. Similarly, we also fitted all models with the predictor “age” instead of “mass” but did not detect any effects and thus used the predictors initially chosen. Before analysis, we removed three data points with extreme values—one in the response variables “dive duration” and “bottom phase duration,” respectively; and two in the predictor “vertical velocity during the descent”—to eliminate possible interference with the models but report these values in the results section. For all response variables, we also included a model with an interaction of the two best predictors in the model selection process. For interactions that included nonzero‐centered predictors, we reran all candidate models with standardized predictors to reduce multicollinearity in the interaction terms (Afshartous & Preston, 2011; Robinson & Schumacker, 2009).

No collinearity between independent variables was detected (*r* < 0.6; apart from water temperature + mass, with *r* = 0.8). However, variance inflation factors (VIF) for all predictors were <3 (Zuur, Ieno, & Elphick, 2010) and we thus included both “temperature” and “mass” as predictors. We applied a backward model selection procedure and selected the most parsimonious models based on the Akaike information criterion (AIC_c_) (Burnham, Anderson, & Huyvaert, 2010; Wagenmakers & Farrell, 2004). We considered candidate models within AIC_c_ differences (ΔAIC_c_) between 0 and 2 as models with strong levels of empirical support (Anderson, 2008) and derived model‐averaged estimates for such models. Model parameters that did not include zero within their 95% confidence interval (CI) were considered as informative (Arnold, 2010). All statistical analyses were performed using the software R 3.3.0. (R Development Core Team 2013).

## 3 RESULTS

We used data from 12 Eurasian beavers (six females and six males) from 11 different territories for analysis (Table 1). In analyses including accelerometry‐based variables, sample size was reduced to 11 individuals, as one accelerometer did not work. Beavers used in analyses had an average mass of 23.3 ± 2.2 kg (Table 1). Individual dataset lengths varied between five full nights (*n* = 1), six full nights (*n* = 5), and seven full nights (*n* = 6). Overall mean values and standard deviations for the dive parameters are presented in Table 2, while the most parsimonious models are presented in Table 3 (for full model selection, see Tables S1–S5); informative predictors and according model outcomes are shown in Table 4.

**Table 1:** Dive analysis metadata for 12 individual Eurasian beavers *Castor fiber* equipped with a data logger measuring pressure and acceleration between 2009 and 2011 in southeast Norway

Beaver	Sex	Mass (kg)	Territory	Year	Month	Water temp (°C)	# Nights	# Dives per night ($\overset{¯}{x}$ )	Max. depth (m) (∼x)	Dive duration (s) (∼x)	Bottom duration (s) (∼x)
Andreas	M	22.5	B1	2010	April	3.1	7	31.3	0.8	19.5	12.8
Chris	M	20.5	L5b	2009	April	3.2	5	35.8	1.0	21.3	10.5
Demi	F	26.9	E	2009	October	9.5	7	35.0	0.7	30.0	18.5
Easy	M	19.5	L5a	2009	April	3.2	6	24.7	1.2	36.0	21.3
Frode	M	22	L2b	2009	May	5.0	6	35.5	0.7	20.6	10.0
Gyda	F	24.2	L4b	2009	May	4.9	6	102.3	0.6	20.0	8.5
Ida	F	23.9	LP	2010	September	10.0	7	94.0	0.6	17.0	12.5
Jan‐Marc	M	23	P0	2011	October	8.7	7	35.9	0.9	21.3	18.3
Kathrin	F	25.5	H	2011	September	11.6	6	56.5	0.9	31.5	17.0
Klumpen	M	26	GM	2009	October	9.4	6	23.3	0.6	27.5	14.5
Leslie	F	22.4	B1	2010	April	3.0	7	19.3	0.7	25.3	10.8
Maud	F	23.8	L6a	2009	November	5.7	7	16.3	0.8	21.3	21.3

**Table 2:** Overall mean values and standard deviations for dive variables derived from 11 Eurasian beavers *Castor fiber* in southeast Norway

Dive variables^a^	($\overset{¯}{x}$)^a^	SD^a^	Unit
Maximum diving depth	0.96	0.60	m
Dive duration	29.24	25.73	s
Mean number of dives per night	39.55	30.97	
Bottom phase duration	14.81	20.72	s
Mean VeDBA during the descent phase	0.18	0.06	g
Mean VeDBA during the bottom phase	0.19	0.07	g
Mean VeDBA during the ascent phase	0.29	0.10	g
Vertical velocity during the descent phase	0.23	0.13	m/s
Vertical velocity during the bottom phase	0.00	0.03	m/s
Vertical velocity during the ascent phase	‐0.13	0.09	m/s

a Calculated using the total amount of values for each variable, not the averaged values for each individual as in Table 1.

**Table 3:** Model selection based on Akaike information criterion corrected for small sample sizes (AICc) for 11 Eurasian beavers *Castor fiber* in southeast Norway. Model averaging was implemented on candidate models within ΔAICc < 2. *w*_i_ = Akaike weight, *K* = Number of parameters, ^#^informative model terms

Response variable	Most parsimonious model	AIC_c_	*w*_i_	*K*
Maximum diving depth	Mean_VeDBA_des^#^ + VV_des^#^	3994.75	0.79	5
Dive duration	VV_des^#^	6083.88	0.69	4
	Mean_VeDBA_des^#^ + VV_des^#^	6085.80	0.27	5
Mean number of dives/night	Water_temp	26.88	0.88	3
Presence of a bottom phase	Max_depth* Mean_VeDBA_des	2418.64	0.45	5
	Max_depth^#^ + Mean_VeDBA_des	2419.88	0.24	4
Bottom phase duration	Max_depth^#^ * Mean_VeDBA_bott^#^	16197.14	0.83	6

Mean_VeDBA_des *Mean VeDBA during the descent phase*; VV_des *Vertical velocity during the descent phase*; Max_diving_depth *Maximum diving depth*; Mean_VeDBA_bott *Mean VeDBA during the bottom phase*.

**Table 4:** Informative model terms for the response variables maximum diving depth, dive duration, and the presence/duration of a bottom phase for 11 Eurasian beavers *Castor fiber* in southeast Norway. All response variables were log‐transformed and numerical predictors scaled, coefficients should thus be interpreted accordingly (for rescaling, see SDs in Table 2). β *= *beta coefficient, σ *= *standard error, LL and UL = lower and upper limits of the 95% confidence interval

Response variable	Informative model term(s)	β	σ	LL	UL
Maximum diving depth	Mean_VeDBA_des	−0.039	0.011	−0.060	−0.018
	VV_des	0.204	0.010	0.184	0.225
Dive duration	Mean_VeDBA_des	−0.011	0.020	−0.073	−0.010
	VV_des	−0.113	0.015	−0.143	−0.082
Presence of a bottom phase	Max_depth	0.575	0.105	0.370	0.781
Bottom phase duration	Mean_VeDBA_bott	−5.807	0.885	−7.283	−4.224
	Max_depth	0.247	0.023	0.203	0.286
	Mean_VeDBA_bott * Max_depth	0.097	0.040	0.015	0.171

Mean_VeDBA_des *Mean VeDBA during the descent phase*; VV_des *Vertical velocity during the descent phase*; Max_diving_depth *Maximum diving depth*; Mean_VeDBA_bott *Mean VeDBA during the bottom phase*.

**Figure 1:**
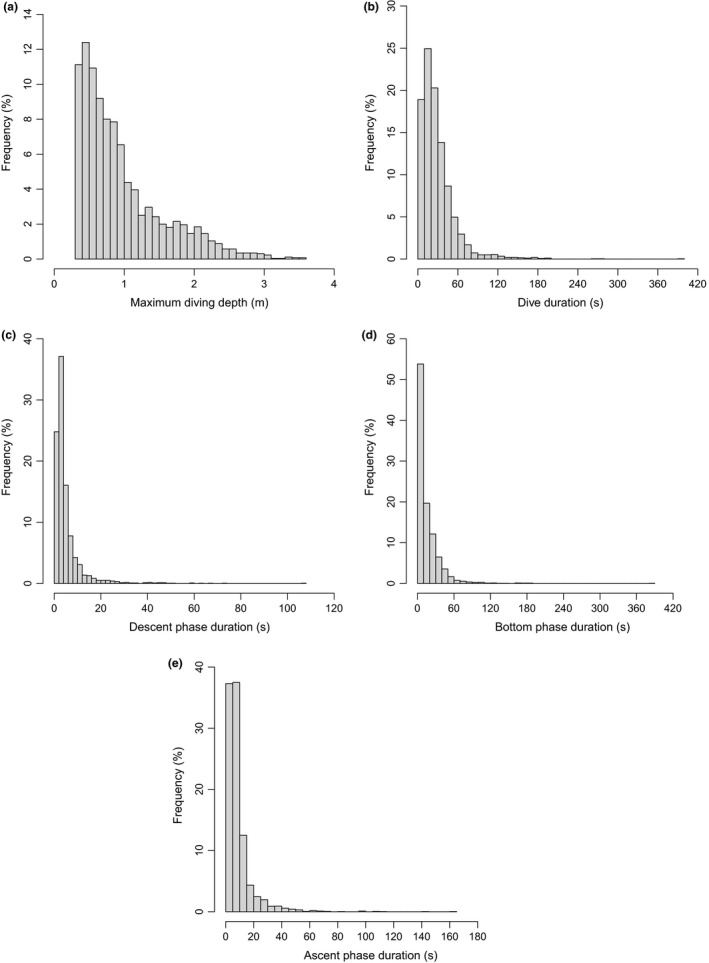
Frequency distributions of maximum diving depth (a), dive duration (b), descent phase duration (c), bottom phase duration (d), and ascent phase duration (e) for 12 Eurasian beavers *Castor fiber* in southeast Norway (*n* = 2596 dives)

**Figure 2:**
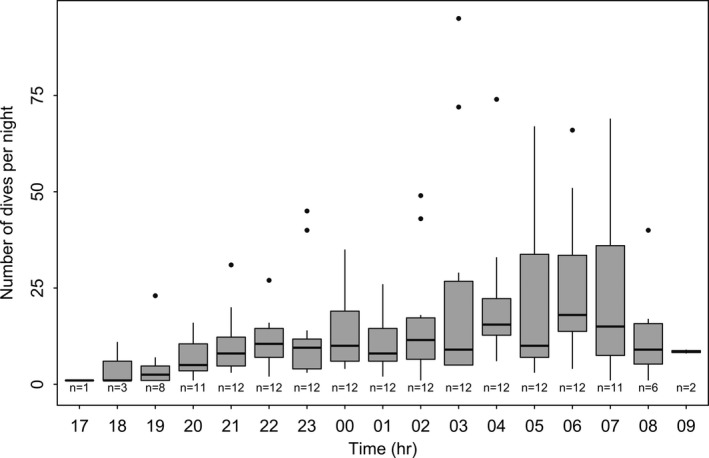
Box‐plot for the number of dives per hour within 5 days (*n* = 2544 dives) for 12 Eurasian beavers *Castor fiber* in southeast Norway. Sample sizes below the boxes depict how many individuals dived at a given hour during their principal activity period

**Figure 3:**
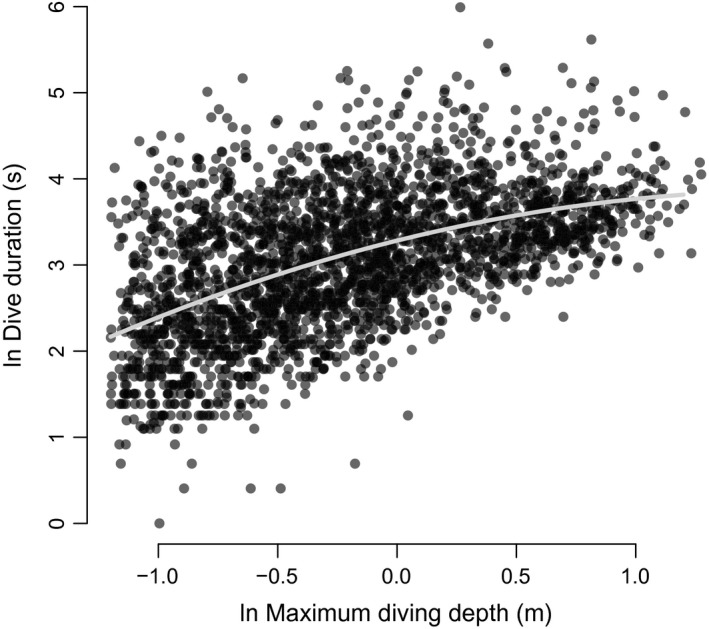
Polynomial regression relationship between dive duration and maximum diving depth in 12 Eurasian beavers *Castor fiber* in southeast Norway (ln dive_dur = 3.283 + 0.687 ln max_depth + −0.203 ln max_depth^2; *F*_2,2595_ *=* 495, *R* ^2^ (adjusted) = 0.27, *p* < .001)

We analyzed a total of 2596 dives, with an overall mean of 39.55 ± 30.97 dives per night (Table 2). Beavers descended with an overall mean VeDBA of 0.18 ± 0.06 *g* (max. 0.64 *g*, Table 2) and an average vertical velocity of 0.23 ± 0.13 m/s (max. 1.88 m/s, Table 2). Overall, mean VeDBA was highest during the ascent phase of a dive ($\overset{¯}{x}$ ± SD = 0.29 ± 0.10 *g*, Table 2). The majority of dives were shallow (<1 m) and short (<30 s) (Tables 1, 2; Figure 1). Diving activities increased throughout the night, with beavers showing a peak diving activity in the latter part of the night, approximately between 03:00 and 07:00 am (Figure 2). Dive duration increased significantly with maximum diving depth, with a polynomial term describing the best fit (β = 0.687, 95% CI = 0.637–0.737, Figure 3). When comparing nightly PAPs (654 ± 63 min) with the total time beavers were submerged each night, we found that diving accounted for only 2.8 ± 2% of the daily activity budget of beavers.

### 3.1 Maximum diving depth

Median “maximum diving depth” for all individuals was 0.77 m, with a range between 0.30 and 3.58 m (overall mean ± *SD* = 0.96 ± 0.60 m, Table 2, Figure 1a). Maximum diving depth was best explained by mean VeDBA and vertical velocity during the descent phase, with the latter relationship being particularly pronounced (Table 3). Higher mean VeDBA values during the descent were associated with shallower maximum diving depths (Table 4), while higher vertical velocities were coupled with deeper maximum diving depths (Table 4).

### 3.2 Dive duration

Dive duration for individual beavers varied between 1 and 400 s with a median of 23 s (overall mean of 29.24 ± 25.73 s, Table 2, Figure 1b). The two best candidate models for dive duration included “vertical velocity during descent,” and both this and “mean VeDBA during descent” (Table 3). We derived model average estimates and their 95% confidence intervals for these two models and found both predictors to be informative (Table 4). Both higher mean VeDBA and vertical velocities during the descent phase resulted in shorter dive durations (Table 4).

### 3.3 Mean number of dives per night

The overall mean number of dives per night was 39.55 ± 30.97 (Table 2). This was best explained by water temperature (Table 3); however, the CI for β estimates incorporated 0 (β = 0.065, LL = −0.039, UL = 0.168), implying that water temperature was an uninformative variable.

### 3.4 Bottom phase duration

We found that 82% of all dives included a bottom phase, which varied between 0.4 and 188 s (median = 12 s) with an overall mean duration of 14.81 ± 20.72 s (Table 2). The decision whether to invest in a bottom phase or not was best explained by two models containing the predictors “mean VeDBA during descent” and “maximum diving depth,” in one model additive, in the other as an interaction (Table 3). We derived averaged model coefficients and their 95% CI and found only maximum diving depth to be informative, so that a bottom phase was more likely in deeper dives (Table 4). When looking at dives with bottom phase only (the GLMM), the best model included the interaction between the predictors “mean VeDBA during the bottom phase” and “maximum diving depth” (Table 3). By inspecting 95% CI, we found “mean VeDBA during the bottom phase” and “maximum diving depth,” as well as the interaction between the two predictors to be informative (Table 4). Higher mean VeDBA values during the bottom phase resulted in shorter bottom phase durations, while diving at greater depths was associated with longer bottom phase durations (Table 4). In addition, the interaction between the two terms implies that bottom durations were also longer in deep dives coupled with high mean VeDBA values (Table 4).

## 4 DISCUSSION

### 4.1 Time spent diving

The predominantly short, shallow dives executed by beavers in our study are similar to a range of other freely diving semi‐aquatic birds and mammals (Snyder, 1983; Thompson & Fedak, 2001). In beavers, muscle oxygen stores deplete after 2–4 min (Snyder, 1983), while blood oxygen stores decrease at 4 min (Clausen & Ersland, 1970), which suggests that our dives were mainly aerobic. Surprisingly, we found that, on average, only 2.8% of the beaver's nightly activity budget comprised diving. This is considerably less than found in other semi‐aquatic species, for example, tufted ducks *Aythya fuligula* dive for 25% of their 24‐hr cycle (Pedroli, 1982) and chick‐rearing Crozet shags *Phalacrocorax melanogenis* for 44% of their at‐sea time (Tremblay, Cook, & Cherel, 2005). In contrast to these species, beavers, as generalist herbivores, do not solely rely on diving for food acquisition (Haarberg & Rosell, 2006). Similarly, American mink, a generalist carnivore, dive for only about 0.5% of the 24‐h cycle (Harrington et al., 2012). Beavers may also have dived less due to (i) the lower availability of aquatic plants in river habitats (Milligan & Humphries, 2010), (ii) the fact that they do not build dams in our study area, a behavior that necessitates diving to seal their dams with mud and stones (Müller & Watling, 2016), and (iii) the lack of data collection during winter (December–March), where the partly ice‐covered rivers may force beavers to dive. Local habitat features and differences in the (seasonal) availability of food resources may thus impact diving behavior in beavers; for example, in pond habitats, beaver diets have been found to contain higher percentages of aquatic vegetation, in particular, during autumn/winter (Milligan & Humphries, 2010).

The niceties of beaver activity patterns explain some of the patterns observed in diving behavior. For example, beavers usually travel along the shoreline (Graf, Mayer, et al., 2016) so the observed short, shallow dives presumably largely reflect the water depths available along the water's edge. Indeed, in terms of foraging, macrophyte growth is encouraged by light penetration and is thus higher at shallow depths (Middelboe & Markager, 1997). This may explain why, even though deeper river sections were present within all territories, no animal dived deeper than 3.6 m. Beyond foraging, other behaviors linked to diving in beavers, such as entering the lodge, submerged transport of sticks/building material, or escaping in the water, do not necessitate particularly deep dives.

Observed behavioral patterns over longer time periods were also reflected in our results. For example, diving activity peaked late during the beaver's PAP although general activity has been found to be highest in the middle of the beaver's PAP (~1 am, see Graf, Hochreiter, et al., 2016). After finishing patrolling their territories (Graf, Mayer, et al., 2016), beavers may have more time to invest in diving activity; similarly, it has been found that building behaviors occur in the latter part of the night (Wilsson, 1971).

### 4.2 Dive parameters and physical effort

**Figure 4:**
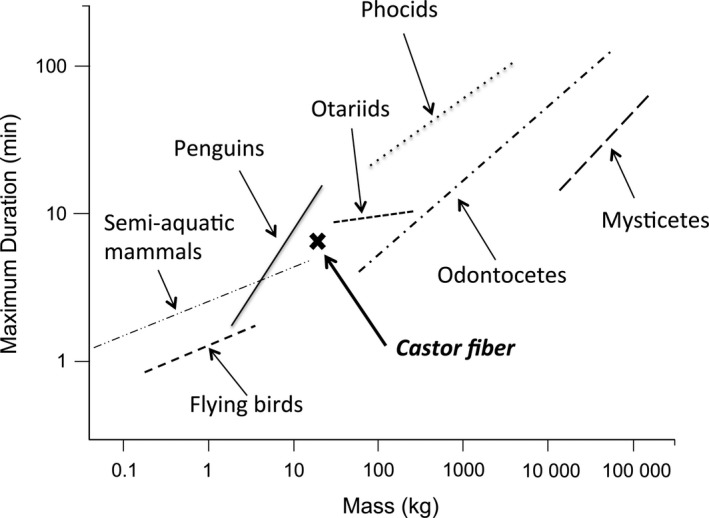
Relationship between maximum dive duration and body mass (both log_10_‐transformed) in a range of animal species and *Castor fiber* (Figure adapted with permission from Schreer & Kovacs, 1997; data on semi‐aquatic mammals included with permission from Harrington et al., 2012)

The allocation of time to depth and the various dive phases by beavers broadly followed patterns established for air‐breathing diving animals. Thus, dive durations increased with greater dive depths (Figure 3), which is a common phenomenon in diving species (e.g., Chilvers, Wilkinson, Duignan, & Gemmell, 2006; Cook, Kato, Tanaka, Ropert‐Coudert, & Bost, 2010). Indeed, the slope estimate ± *SE* of 0.69 ± 0.03 for beavers compares well with other diving birds and mammals (c.f. Schreer & Kovacs, 1997; Halsey, Blackburn, et al., 2006; Figure 4). Thus, in this respect, semi‐aquatic beavers tie in with more aquatic species. In addition though, beavers are heavier than most semi‐aquatic species and diving birds (Fish, 2000; Harrington et al., 2012), so longer dive durations (Figure 4) can likely be attributed to their higher body mass. This is because oxygen storage scales as a function of mass to the power of one, while metabolic rate scales with mass to the power of 0.67 so smaller animals use their available oxygen stores relatively faster (Halsey, Blackburn, et al., 2006). In short, overall, looking at the relationship between dive duration and body mass, beavers appear to conform absolutely to that expected for the more specialized divers (Figure 4).

Some of the allocation of effort to various phases of the dives, however, fitted less well. In the first instance though, VeDBA, taken as a proxy for movement power (Qasem et al., 2012), was highest near the surface for the descending animals, as observed in a number of air‐breathing diving homeotherms (Wilson, Shepard, Laich, Frere, & Quintana, 2010; Wilson et al., 2006). This is presumed to be due to the fur‐ and lung‐associated air (Fish et al., 2002; McKean & Carlton, 1977), which has maximum volume and highest upthrust closest to the surface. This is particularly the case in diving birds, which have large amounts of plumage‐entrapped air and have to use greatest power to overcome this upthrust closest to the surface (Wilson et al., 1992, 2010). Increasing depth changes this though because air is compressed with depth following Boyle's Law.

$$P_{1}V_{1}=P_{2}V_{2}$$

where *P*_1_ and *P*_2_ are the pressures (in Bar) at the surface and depth, respectively, while *V*_1_ and *V*_2_ are the volumes of air at the respective depths. Thus, in the first meter alone, the volume of any animal‐associated air decreases by some 10% with an expected corresponding decrease in power required to counteract upthrust (c.f. Wilson et al., 1992). In addition, within the first moments of a dive, extra power is expected to accelerate the animal from a vertical speed of 0 m/s (at the surface) to the normal descent speed as well as to counteract surface drag (Williams, 2001; Wilson et al., 1992).

Given the compression of air with depth and its apparent explanation for what we observed during the descent phases of beavers’ dives, we expected the reverse process of power allocation to occur during the ascent phases specifically that VeDBAs should be lower as buoyancy pushed the animals to the surface (c.f. Wilson et al., 2010). Curiously though, ascent was the most VeDBA‐intense moment of the dive (Table 2). We have three possible explanations for this (i) that beavers may often surface transporting plants and other materials, and so have to increase power to contend with the increased drag or (ii) that the air in the pelage may bubble out with time underwater. Supporting this, threatened beavers have been observed to remain motionless at depth (personal communication, F. Rosell) when, if the air remained, they would be expected to float to the surface (c.f. Sato, Aoki, Watanabe, & Miller, 2013). Finally, (iii) shallow‐diving animals may increase dive duration by expelling air from their lungs to get them to neutral buoyancy (Wilson et al., 1992). The reduced oxygen availability may then be compensated by the energy otherwise used to swim against positive upthrust. Indeed, such a scenario has been proposed for cormorants and is only possible because their overall body density is close to that of water (Wilson et al., 1992). Given the extremely low profile of surface‐swimming beavers, they must be in this bracket although further work is needed to clarify these issues.

Beavers, such as birds, showed an increase in descent vertical velocity with increasing diving depth (Cook et al., 2010; Noda, Kikuchi, Takahashi, Mitamura, & Arai, 2016). Indeed, for most diving animals, deeper dives are generally accompanied by steeper dive angles (Ropert‐Coudert et al., 2001), so, even with constant swim speed, the rate of change in depth is higher. Sato, Charrassin, Bost, and Naito (2004) suggest that this helps animals maximize bottom duration by reducing the transit time, but it is actually power use that determines how fast body oxygen stores deplete. This means that shorter, but higher power, descent dive phases can actually reduce oxygen available for the bottom phase (Wilson et al., 2006). Indeed, this phenomenon alone may explain why higher descent vertical velocities tended to be associated with shorter dive durations. With regard to the bottom phase, extended durations generally accompanied lower mean VeDBA values. Given that VeDBA is a proxy for power, this is entirely expected. Nonetheless, there were exceptions to this, with some deeper dives incurring high mean VeDBA values during the bottom phase and longer bottom phase durations. We suggest that such phenomena may be linked to a specific dive type, for example, foraging for aquatic plants, and may result in some dives being anaerobic.

### 4.3 Water temperature

A major factor that is reputed to affect diving costs in aquatic homeotherms is water temperature (Bevan & Butler, 1992; Ciancio et al., 2016) and, correspondingly, reduced diving behavior during winter has been found in both muskrats (MacArthur, 1984) and star‐nosed moles *Condylura cristata* (McIntyre, Campbell, & MacArthur, 2002). In beavers, body temperature has been found to drop significantly during swimming and, in particular, in winter (Nolet & Rosell, 1994; Smith, Peterson, Drummer, & Sheputis, 1991). The thermoneutral zone of beavers lies between 0–2°C and 28°C in air (MacArthur & Krause, 1989), although in water, beavers display abdominal cooling already at water temperatures between 2 and 20°C (MacArthur & Dyck, 1990). It is therefore curious that water temperature did not influence diving behavior.

However, beavers have a suite of specialized morphological (thick fur, adipose tissue; Novak, 1987), physiological (e.g., bradycardia, peripheral vasoconstriction, local heterothermy, Swain et al., 1988; MacArthur & Dyck, 1990), and behavioral adaptations that enable them to operate in cold water. The latter may include spending more time on land or inside the lodge (Nolet & Rosell, 1994; Smith et al., 1991) or the selection of food rich in polyunsaturated fatty acids, which improve membrane functionality at low temperatures (Hazel, 1995) and comprise a major part of the adipose tissues of beavers (Zalewski, Martysiak‐Żurowska, Chylińska‐Ptak, & Nitkiwicz, 2009). Ultimately, beavers may also dive to access their food cache under the ice in winter (Dyck & MacArthur, 1992), although the lack of winter diving data impedes further conclusion in this regard.

## 5 CONCLUSION

Beavers were similar to other semi‐aquatic freshwater divers and executed mainly short (likely aerobic) and shallow dives. However, as they are not obliged to dive for food, they dived markedly less than many other semi‐aquatic species (2.8% of their active time). Physical effort during the dive phases and diving depth described the studied dive parameters and shed light on the beaver's strategies to optimize diving behavior by counterbalancing diving costs related to buoyancy, drag, and limited oxygen supply. Ultimately, physiological measurements including heart rate and body temperature of diving individuals should be combined with behavioral research to deepen our understanding of diving tactics and dive responses in semi‐aquatic homeotherms.

## CONFLICT OF INTEREST

None declared.

## AUTHOR CONTRIBUTIONS

PMG, FR, RPW, and KH conceived the ideas and designed the study; PMG, FR, and LCS collected and analyzed the data; PMG, RPW, FR, and LCS interpreted the results; PMG, RPW, FR, KH, and LCS wrote the paper. All authors contributed critically to the drafts and gave final approval for publication.
