# Supplementary material for: Diving behavior in a free‐living, semi‐aquatic herbivore, the Eurasian beaver Castor fiber
Source: Ecol Evol. 2017 Dec 12;8(2):997–1008. doi: 10.1002/ece3.3726 (PMC5773300; doi:10.1002/ece3.3726)
Supplement: Supplementary file 1 [file ECE3-8-997-s001.docx]

**Table S1.** Model selection using Akaike’s information criterion corrected for small sample sizes (AIC_c_) for maximum diving depth in relation to the five predictor variables for 11 dominant Eurasian beavers (*Castor fiber*) in Telemark, southeastern Norway (2009 – 2011). ΔAICc = Difference in AICc relative to min. AICc; w_i_ = Akaike weight, K = Number of parameters

| **Maximum diving depth** |  |  |  |  |  |
| --- | --- | --- | --- | --- | --- |
| Model | AIC_c_ | ΔAIC_c_ | w_i_ | K | Deviance |
| mean_VeDBA_des + VV_des | 3994.75 | 0.00 | 0.79 | 5 | -1992.36 |
| VV_des | 3998.37 | 3.62 | 0.13 | 4 | -1995.18 |
| mean_VeDBA_des + VV_des + weight | 3999.54 | 4.79 | 0.07 | 6 | -1993.76 |
| mean_VeDBA_des * VV_des | 4003.56 | 8.81 | 0.01 | 6 | -1995.76 |
| mean_VeDBA_des + VV_des + weight + water_temp | 4005.19 | 10.44 | 0.00 | 7 | -1995.57 |
| mean_VeDBA_des + VV_des + weight + water_temp + sex | 4010.26 | 15.51 | 0.00 | 8 | -1997.10 |
| mean_VeDBA_des | 4348.75 | 354 | 0.00 | 4 | -2170.37 |

mean_VeDBA_des = mean VeDBA during the descent, VV_des = vertical velocity during the descent, water_temp =
water temperature

**Table S2.** Model selection using Akaike’s information criterion corrected for small sample sizes (AIC_c_) for dive duration in relation to the five predictor variables for 11 dominant Eurasian beavers (*Castor fiber*) in Telemark, southeastern Norway (2009 – 2011). ΔAICc = Difference in AICc relative to min. AICc; w_i_ = Akaike weight, K = Number of parameters

| **Dive duration** |  |  |  |  |  |
| --- | --- | --- | --- | --- | --- |
| Model | AIC_c_ | ΔAIC_c_^b^ | w_i_^c^ | K^d^ | Deviance |
| VV_des | 6083.88 | 0.00 | 0.69 | 4 | -3037.93 |
| mean_VeDBA_des + VV_des | 6085.80 | 1.91 | 0.27 | 5 | -3037.89 |
| mean_VeDBA_des * VV_des | 6090.13 | 6.24 | 0.03 | 6 | -3039.05 |
| mean_VeDBA_des + VV_des + weight | 6091.95 | 8.06 | 0.01 | 6 | -3039.96 |
| mean_VeDBA_des + VV_des + weight + water_temp | 6096.74 | 12.86 | 0.00 | 7 | -3041.35 |
| mean_VeDBA_des + VV_des + weight + water_temp + sex | 6100.79 | 16.91 | 0.00 | 8 | -3042.37 |
| mean_VeDBA_des | 6129.69 | 45.81 | 0.00 | 4 | -3060.84 |

mean_VeDBA_des = mean VeDBA during the descent, VV_des = vertical velocity during the descent, water_temp =
water temperature

**Table S3.** Model selection using Akaike’s information criterion corrected for small sample sizes (AIC_c_) for the mean number of dives per night in relation to the three predictor variables for 12 dominant Eurasian beavers (*Castor fiber*) in Telemark, southeastern Norway (2009 – 2011). ΔAICc = Difference in AICc relative to min. AICc; w_i_ = Akaike weight, K = Number of parameters

| **Mean number of dives per night** |  |  |  |  |  |
| --- | --- | --- | --- | --- | --- |
| Model | AIC_c_ | ΔAIC_c_^b^ | w_i_^c^ | K^d^ | Deviance |
| water_temp | 26.88 | 0.00 | 0.88 | 3 | -8.94 |
| water_temp + sex | 31.07 | 4.18 | 0.11 | 4 | -8.68 |
| water_temp * sex | 36.03 | 9.14 | 0.01 | 5 | -8.01 |
| water_temp + sex + weight | 36.70 | 9.82 | 0.01 | 5 | -8.35 |

water_temp = water temperature

**Table S4.** Model selection using Akaike’s information criterion corrected for small sample sizes (AIC_c_) for the decision to invest in a bottom phase in relation to the three predictor variables for 11 dominant Eurasian beavers (*Castor fiber*) in Telemark, southeastern Norway (2009 – 2011). ΔAICc = Difference in AICc relative to min. AICc; w_i_ = Akaike weight, K = Number of parameters

| **Investment in bottom phase** |  |  |  |  |  |
| --- | --- | --- | --- | --- | --- |
| Model | AIC_c_ | ΔAIC_c_^b^ | w_i_^c^ | K^d^ | Deviance |
| mean_VeDBA_des * max_depth | 2418.64 | 0.00 | 0.45 | 5 | -1204.31 |
| mean_VeDBA_des + max_depth | 2419.88 | 1.24 | 0.24 | 4 | -1205.93 |
| max_diving_depth | 2420.65 | 2.01 | 0.17 | 3 | -1207.32 |
| mean_VeDBA_des + max_depth + water_temp | 2421.06 | 2.41 | 0.14 | 5 | -1205.52 |
| mean_VeDBA_des | 2451.31 | 32.66 | 0.00 | 3 | -1222.65 |

mean_VeDBA_des = mean VeDBA during the descent, max_depth = maximum diving depth, water_temp =
water temperature

**Table S5.** Model selection using Akaike’s information criterion corrected for small sample sizes (AIC_c_) for bottom phase duration in relation to the three predictor variables for 11 dominant Eurasian beavers (*Castor fiber*) in Telemark, southeastern Norway (2009 – 2011). ΔAICc = Difference in AICc relative to min. AICc; w_i_ = Akaike weight, K = Number of parameters

| **Bottom phase duration** |  |  |  |  |  |
| --- | --- | --- | --- | --- | --- |
| Model | AIC_c_ | ΔAIC_c_^b^ | w_i_^c^ | K^d^ | Deviance |
| mean_VeDBA_bott * max_depth | 16197.14 | 0.00 | 0.83 | 6 | -8092.55 |
| mean_VeDBA_bott + max_depth | 16201.11 | 3.97 | 0.11 | 5 | -8095.54 |
| mean_VeDBA_bott + max_depth + water_temp | 16202.73 | 5.60 | 0.05 | 6 | -8095.35 |
| max_diving_depth | 16263.21 | 66.07 | 0.00 | 4 | -8127.59 |
| mean_VeDBA_bott | 16323.17 | 126.03 | 0.00 | 4 | -8157.57 |

mean_VeDBA_bott = mean VeDBA during the bottom phase, max_depth = maximum diving depth, water_temp
= water temperature
